# Analysis of *P* Values in the Abstract Compared With the Main Text of Randomized Controlled Trials and Clinical Trials of Mesenchymal Stromal Cells for the Treatment of Knee Osteoarthritis

**DOI:** 10.1177/23259671251374306

**Published:** 2025-10-15

**Authors:** Nesa Milan, Katherine Woolley, Zubin Master, Brian T. Feeley

**Affiliations:** * Department of Orthopaedic Surgery, University of California, San Francisco, San Francisco, California, USA; † Department of Social Sciences and Health Policy, Wake Forest University, Winston-Salem, North Carolina, USA; ‡ Wake Forest Institute for Regenerative Medicine, Wake Forest University, Winston-Salem, North Carolina, USA; § Maya Angelou Research Center for Healthy Communities, Wake Forest University, Winston-Salem, North Carolina, USA; ‖ Center for Bioethics, Health, and Society, Wake Forest University, Winston-Salem, North Carolina, USA; Investigation performed at the Department of Orthopaedic Surgery, University of California, San Francisco, San Francisco, California, USA

**Keywords:** MSCs, knee osteoarthritis, abstract, bias

## Abstract

**Background:**

Randomized controlled trials (RCTs) and clinical trials (CTs) are critical for evaluating treatment effectiveness, with abstracts serving as the primary source of the study that is read.

**Purpose:**

This study explored selective reporting of significant *P* values in the abstracts of RCTs and CTs on mesenchymal stromal cells (MSCs) for the treatment of knee osteoarthritis (OA). The purpose was to compare the rates of significant *P* values reported in the abstract compared with the main text of RCTs and CTs on MSCs for the treatment of knee OA.

**Study Design:**

Systematic review.

**Methods:**

PubMed (MEDLINE) and Embase searches were conducted using related terms and were filtered for RCTs and CTs. There were 2 reviewers who analyzed the abstract and main text independently and extracted study characteristics and every *P* value reported in the abstract and main text of each article. The distribution of significant *P* values between the abstract and main text was compared using a paired-samples *t* test and odds ratios. Significance was set at *P* < .05.

**Results:**

The initial search yielded 156 articles, with 61 meeting the inclusion criteria. Our analysis showed a significant discrepancy in the distribution of *P* values between the abstract and main text. In abstracts, 79.3% of *P* values were significant compared with 53.8% in main texts. The odds of significant *P* values being reported in the abstract over the main text were more than 3 times higher (odds ratio, 3.3). The paired-samples *t* test confirmed that abstracts were significantly more likely to report significant findings (*P* = 7.5 × 10^−8^). Primary outcomes were reported in 90.2% of abstracts, with 18.0% being significant, 47.5% being not significant, and 34.4% being not applicable because of qualitative results. Functional and quality of life scores were the most common primary outcomes in 34 studies, followed by safety and adverse events in 25 studies, pain measurements in 20 studies, and imaging outcomes in 17 studies.

**Conclusion:**

Our study highlights a significant risk of reporting bias in RCTs and CTs of MSCs for the treatment of knee OA. With growing interest in the use of MSCs, it remains crucial for busy clinicians to recognize the possibility of selective reporting bias in abstracts, as this could potentially influence clinical decision making.

Knee osteoarthritis (OA) is a common condition involving degeneration of the joint with cartilage loss and bony changes.^33^ Mesenchymal stromal cells (MSCs; also known as mesenchymal stem cells) can differentiate into mesodermal lineages such as osteocytes and chondrocytes. The use of MSCs for the treatment of musculoskeletal conditions has garnered significant attention for their pluripotent potential, including increased exploration of their clinical utility in treating knee OA.^21,30,32^ The burgeoning interest is evidenced by accelerating rates of research on biologics for cartilage repair and OA treatment, with rates increasing by 16.4% annually from 2006 to 2021.^35^ This has not only sparked interest in the medical community but has also gained the attention of patients who seek clinical consultations and conduct online searches for stem cell injections to treat hip and knee OA.^3,31^ Despite increased public and research interest on the topic, systematic reviews of biologics for the treatment of OA have found that studies are at risk of bias, with small numbers of patients and the lack of a control and randomization.^1,13^

Randomized controlled trials (RCTs) and clinical trials (CTs) are the gold standard for evaluating the effectiveness of treatment methods or interventions and are paramount for making evidence-based medical treatment decisions.^4,16,18^ Abstracts provide a summary of study findings and are essential for efficiently conveying critical results.^19^ Beyond article titles, abstracts are the main and frequently sole section of an article that is read, highlighting the critical findings of studies; therefore, accurate and complete reporting is vital as clinicians and researchers often make important judgments based only on information from abstracts.^5,18,24,25^ Previous studies have investigated the bias and quality of data reported in abstracts. Spin bias,^34^ or reporting that could alter the interpretation of results and misguide readers, has been examined in several fields, including orthopaedics.^7^ Studies investigating spin in systematic reviews and meta-analyses of treatment for lumbar disc herniation found spin in 29.4% of abstracts, another found spin in 35.4% of abstracts for the surgical treatment of knee OA, and finally, spin was found in 65.1% of abstracts on treatment for Achilles tendon ruptures.^2,8,29^ Selective reporting was the most common form of spin found by the latter 2 studies.^8,29^ Selective reporting, a form of bias in which only the most favorable results are highlighted, is often observed in abstracts, potentially misrepresenting the full findings of the study. This issue is particularly concerning in knee OA research. To address this, Assem et al^4^ explored a way to identify selective reporting in abstracts by comparing the proportion of significant *P* values in the abstract with those in the main text. To our knowledge, no study has investigated selective reporting in the abstracts of RCTs and CTs on MSCs for the treatment of knee OA. This study aimed to compare the rates of significant *P* values reported in the abstract compared with the main text of these studies and to identify trends in reported outcomes. We hypothesized that the abstracts of RCTs and CTs involving MSCs for the treatment of knee OA would show a higher proportion of significant *P* values compared with the corresponding main text. This may suggest the presence of selective reporting bias in which more significant results are highlighted in the abstracts than in main texts. This analysis addresses a gap in the literature by focusing on MSC-based interventions for knee OA, a rapidly expanding area of research in which abstract-level reporting may influence how findings are interpreted and communicated.

## Methods

### Article Selection

**Figure 1.**
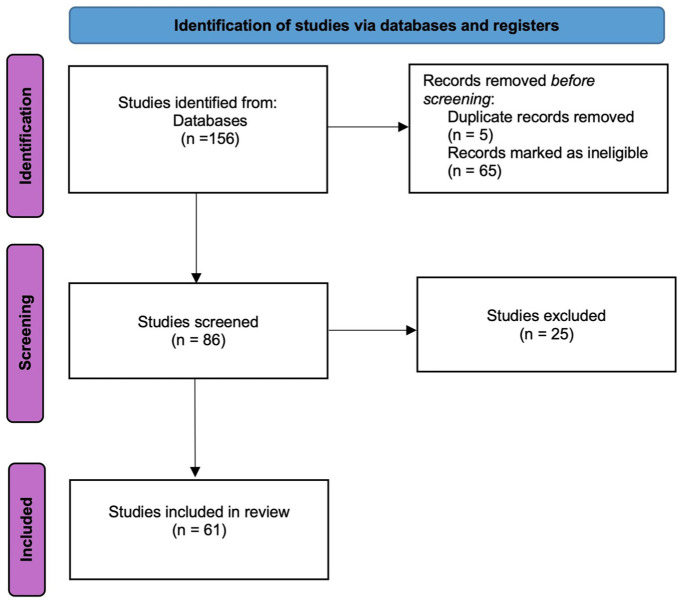
Flow diagram.

Our study utilized a systematic review approach, involving a comprehensive literature search, study selection, data extraction, and qualitative synthesis following PRISMA (Preferred Reporting Items for Systematic Reviews and Meta-Analyses)^23^ guidelines (Figure 1). This study examined RCTs and CTs of knee OA treated with MSCs for selective reporting of significant *P* values. PubMed (MEDLINE) and Embase searches were conducted on July 23, 2023, using the terms “mesenchymal stem cells” or “mesenchymal stromal cells” or “MSCs” and “knee arthritis” or “osteoarthritis” and “therapy” or “treatment” or “regeneration.” Studies were included if they were a prospective RCT or CT involving human participants, MSCs, and primary knee OA and if the full article was available in English. Studies were uploaded to a datasheet, and duplicates were removed. All articles were screened independently for inclusion by 2 reviewers (N.M. and K.W.). Both reviewers agreed on the inclusion or exclusion of studies.

### Data Extraction

The abstract and main text of studies meeting the inclusion criteria were analyzed independently (N.M. and K.W.), and data collected included the following: number of significant *P* values in the abstract and text, total number of reported *P* values in the abstract and text, journal/book, publication year, intervention, control, primary outcomes, secondary outcomes, impact factor, and financial disclosures. Statistical significance was determined by the article and reported *P* value, with significance at *P* < .05. Any outcomes without data for statistical significance were not included in the analysis. The measured outcomes were further categorized as follows.

Pain measurements: This included the Numeric Pain Rating Scale, visual analog scale, Western Ontario and McMaster Universities Osteoarthritis Index (WOMAC) pain subscale, Knee Injury and Osteoarthritis Outcome Score (KOOS) pain subscale, and other specific pain measures such as pain on walking and number of stairs until pain appears.
Functional and quality of life scores: This encompassed the KOOS (excluding the pain subscale), WOMAC (excluding the pain subscale), Lequesne algofunctional index, 12-item Short Form Health Survey, International Knee Documentation Committee form, Lysholm score, Oxford Knee Score, and Numeric Rating Scale–11.
Imaging outcomes: This included magnetic resonance imaging (MRI), radiography, and specific MRI scores such as the Whole-Organ MRI Score, T2 values for cartilage morphology and collagen content, serial changes in the cartilage defect, International Cartilage Repair Society grade, Magnetic Resonance Observation of Cartilage Repair Tissue, hip-knee-ankle angle, and Kellgren-Lawrence grade.
Safety and adverse events: This category included adverse events, severe adverse events, tolerability, and specific safety assessments such as laboratory tests, vital signs, physical examinations, and specific criteria such as the Common Terminology Criteria for Adverse Events (Version 4.0).
Specific clinical scores and indices: This encompassed the Knee Society Score, need for total hip replacement, and clinical failure rate.
Miscellaneous outcomes: This included other specific outcomes mentioned such as range of motion, patellar crepitus, presence of synovial fluid, and bone marrow aspirate concentrate characterization.

Coding was performed independently by the 2 reviewers (N.M. and K.W.), who alternated codes on half of the studies, and flagged audits were performed throughout the review process by the same 2 coders to ensure that assessments were reproducible. A third reviewer (B.T.F.) performed random audits of 10 studies (each coded by N.M. and K.W.), and finally, one of the reviewers (K.W.) then appraised the entire database.

### Statistical Analysis

We conducted a statistical analysis to compare the distribution of statistically significant *P* values (*P* < .05) between abstracts and main texts. A paired-samples *t* test was used for this comparison. Additionally, odds ratios (ORs) were calculated to assess the likelihood of encountering significant *P* values in the abstract versus main text. Assem et al^4^ provided a methodological reference for identifying selective reporting in abstracts. Their approach involved calculating the OR to analyze the association between significant *P* values and what was reported in the abstract. This comparison allows us to assess the presence of reporting bias in surgical RCTs. Descriptive statistics were utilized to summarize the frequency of primary outcome categories reported in the reviewed studies. All analyses were conducted using Python (Version 3.7), with statistical significance set at *P* < .05.

## Results

**Figure 2.**
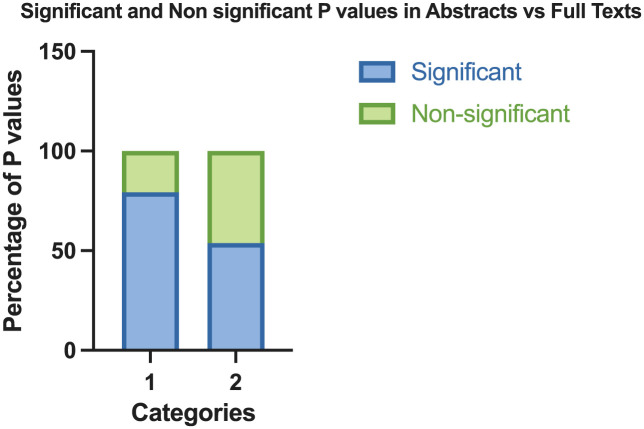
The frequency of significant (*P* < .05) and insignificant (*P* > .05) *P* values reported in (1) abstracts versus (2) main texts.

The initial search returned 156 articles, with 61 meeting inclusion criteria. Our analysis of the distribution of statistically significant *P* values between the abstract and main text of articles revealed a significant discrepancy. A total of 213 *P* values were reported in the abstract, with 169 (79.3%) being significant and 44 (20.7%) being insignificant. In comparison, a total of 1935 *P* values were reported in the main text, with 1041 (53.8%) being significant and 894 (46.2%) being insignificant (Figure 2). OR analysis showed that the abstract was over 3 times more likely to report significant *P* values compared with the main text (OR, 3.299). The paired-samples *t* test comparing the distribution of significant and nonsignificant *P* values in abstracts and main texts highlighted the differences in reporting, with abstracts significantly more likely to report significant findings (*P* = 7.47 × 10^−8^).

**Figure 3.**
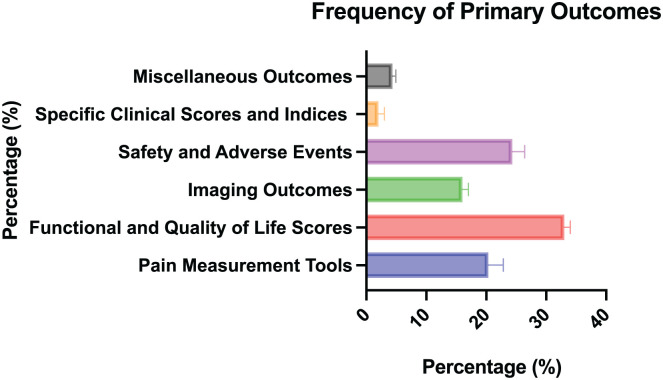
Frequency of primary outcomes in abstracts.

Primary outcomes were reported in 55 (90.2%) abstracts and not reported in 6 (9.8%) abstracts. Among the studies with reported primary outcomes, 40 were registered, and 15 were not. Among the articles analyzed, 11 (18.0%) reported significant primary outcomes, 29 (47.5%) found their primary outcomes to be not significant, and 21 (34.4%) were categorized as “not applicable” because of the primary outcomes being qualitative and without statistical analysis such as safety and adverse events. Functional and quality of life scores were the most reported primary outcomes in 34 studies, followed by safety and adverse events in 25, pain measurements in 20, and imaging outcomes in 17 (Figure 3 and Table 1). The mean abstract word count was 285 words, and there was no association between the abstract word count and the proportion of significant *P* values (*P* > .05).

**Table 1:** Primary Outcomes in Abstracts

	n (%)
Pain measurements	20 (19.61)
Functional and quality of life scores	34 (33.33)
Imaging outcomes	17 (16.67)
Safety and adverse events	25 (24.51)
Specific clinical scores and indices	2 (1.96)
Miscellaneous outcomes	4 (3.92)

## Discussion

Abstracts were over 3 times more likely to report significant *P* values compared with main texts. Additionally, our investigation into primary outcomes revealed that while 90.2% of abstracts reported primary outcomes, they were absent in 9.8% of them. Our research underscores a notable inconsistency in how *P* values are reported between the abstract and main text of RCTs and CTs focused on MSC therapy for knee OA. This discrepancy could potentially misguide readers who primarily consult abstracts for rapid evaluations of the literature, thereby fostering a biased understanding of the treatment's efficacy.

Because of the limited space in abstracts, authors might prioritize primary outcomes, which are more likely to show significance, or focus on presenting the most clinically relevant findings that could influence practice. Thus, secondary outcomes and nonsignificant results may be underreported or omitted, potentially leading to an incomplete understanding of the study's overall findings and their broader implications. A review revealed that CTs with positive findings were 3.9 times more likely to be published than those with negative or nonsignificant results.^17^

Lehmen et al^20^ identified substantial discrepancies between abstracts and main texts, noting inconsistencies in 75% of the lumbar spine surgery studies that they reviewed. Notably, only 60% of the abstracts reported relevant statistically significant results. Their analysis revealed that important details, such as methods of randomization and primary outcomes, were often missing or poorly described in abstracts.^9-11,20^ Pocock et al^27^ pointed out that CTs often include vast amounts of data, making it difficult to interpret results accurately, especially with excessive significance testing. They suggested a more transparent approach in which primary treatment comparisons are predefined and greater emphasis is placed on the actual size of treatment effects and the use of confidence intervals rather than arbitrary significance thresholds.^27^ Confidence intervals are better because they provide a range of values within which the true effect size is likely to lie, offering more information about the precision and reliability of the estimated effect. This approach avoids the binary interpretation of results as simply “significant” or “not significant,” which can be misleading. *P* values in observational research can be especially deceptive and should not be interpreted as probabilities. This core problem likely explains why *P* values in cohort studies tend to be the most extreme, as data from large cohorts are frequently published repeatedly.^15,26^

Selective reporting, as evidenced in this study, can significantly affect clinical decision making. Specifically, abstracts were found to contain a higher proportion of significant *P* values compared with their corresponding main texts. This is similar to what Rongen and Hannink^28^ discovered when they examined the consistency between registered and published primary outcomes in orthopaedic surgical RCTs. They found discrepancies in 53.8% of the trials, usually favoring significant results. Such selective reporting undermines the validity of evidence-based medicine by increasing false-positive results and potentially misleading health care professionals and policymakers.^10,28^ Such reporting may lead clinicians and researchers to develop a biased view of the effectiveness of MSC therapy for knee OA, affecting treatment recommendations and future research directions. This study underscores the need for careful and critical appraisal of published data, advocating for a thorough evaluation of the entire text rather than relying solely on the abstract. Research reporting general health measurements should carefully consider power calculations, as underpowered secondary outcomes may fail to reach statistical significance, despite potential clinical relevance. This can contribute to selective reporting in abstracts in which well-powered primary outcomes are emphasized while secondary outcomes remain underreported. Recognizing this issue helps to provide a more balanced interpretation of study findings. Numerous studies have warned against relying solely on abstracts, as it could mislead readers, particularly those who depend only on abstracts for quick assessments of the scientific literature, thereby perpetuating a biased interpretation of the treatment's efficacy.^6,14^ Our findings emphasize the need for a careful and critical appraisal of published data. Utilizing the full medical literature for clinical decisions has been shown to be more effective.^12,22^

Our study had several limitations. We did not evaluate the quality of the individual studies or compare journals based on their quality metrics. Comparing journals based on established quality indicators could provide more insight into the impact of journal quality on research findings. Future directions could involve the development of stricter guidelines for abstract reporting and the implementation of checks that ensure consistency across abstracts and main texts.

## Conclusion

While abstracts are a convenient way to access scientific information, the current findings emphasize the risk of relying solely on them for understanding a study's full context. Alternatively, authors might aim to attract readers or journal editors by presenting an increased number of significant findings. With a substantial discrepancy observed in the prevalence of reported significant *P* values between the abstract and main text, our study accentuates the potential for abstracts to misrepresent research findings. This bias not only affects clinical judgment but also distorts the scientific narrative surrounding effective interventions for knee OA. Our findings advocate for enhanced editorial oversight and the encouragement of transparent reporting practices.
